# Coherent Domains of Transcription Coordinate Gene Expression During Bacterial Growth and Adaptation

**DOI:** 10.3390/microorganisms7120694

**Published:** 2019-12-13

**Authors:** Georgi Muskhelishvili, Raphaël Forquet, Sylvie Reverchon, Sam Meyer, William Nasser

**Affiliations:** 1 Agricultural University of Georgia, 0159 Tbilisi, Georgia; g.muskhelishvili@icloud.com; 2 INSA-Lyon, CNRS, UMR5240, Microbiologie, Adaptation, Pathogénie, Univ. Lyon, Université Lyon 1, F-69622 Villeurbanne, France; raphael.forquet@insa-lyon.fr (R.F.); sylvie.reverchon-pescheux@insa-lyon.fr (S.R.); sam.meyer@insa-lyon.fr (S.M.)

**Keywords:** bacteria, chromosomal origin-to-terminus axis, DNA thermodynamic stability, DNA supercoiling, nucleoid-associated proteins, transcription, chromosomal domains, genetic regulation

## Abstract

Recent studies strongly suggest that in bacteria, both the genomic pattern of DNA thermodynamic stability and the order of genes along the chromosomal origin-to-terminus axis are highly conserved and that this spatial organization plays a crucial role in coordinating genomic transcription. In this article, we explore the relationship between genomic sequence organization and transcription in the commensal bacterium *Escherichia coli* and the plant pathogen *Dickeya.* We argue that, while in *E. coli* the gradient of DNA thermodynamic stability and gene order along the origin-to-terminus axis represent major organizational features orchestrating temporal gene expression, the genomic sequence organization of *Dickeya* is more complex, demonstrating extended chromosomal domains of thermodynamically distinct DNA sequences eliciting specific transcriptional responses to various kinds of stress encountered during pathogenic growth. This feature of the *Dickeya* genome is likely an adaptation to the pathogenic lifestyle utilizing differences in genomic sequence organization for the selective expression of virulence traits. We propose that the coupling of DNA thermodynamic stability and genetic function provides a common organizational principle for the coordinated expression of genes during both normal and pathogenic bacterial growth.

## 1. Introduction

Coordination of the bacterial chromosome structure and function is mediated by changes in genomic DNA supercoiling associated with DNA replication and its cessation and corresponding alterations of transcription. The mechanism coordinating genomic transcription is not fully understood, but recent findings made it increasingly clear that the genetic regulation of bacterial growth cannot be satisfactorily explained on the basis of the classical operon model. First of all, genomic transcription turned out to be both pervasive and fuzzy, so that the operon model does not hold the promise to explain all the alterations of genetic activity during bacterial growth and adaptation, as thought before [1,2,3,4,5,6]. Secondly, positional effects on reporter gene expression were observed in *Escherichia coli* [7,8,9], consistent with the notion that genetic expression not only is regulated in digital (discontinuous) mode—that is, by classical transcription factor (TF)–target gene (TG) interactions—but also is subject to the analog (continuous) mode of control, mediated by cell physiology-dependent changes of chromosomal DNA supercoiling [4,10,11,12,13,14,15]. Furthermore, recent studies discovered a local impact of transcription-coupled supercoil diffusion (TCSD) on transcription initiation from nearby promoters [16,17,18], whereas high-throughput studies discovered coherent domains of transcription spanning chromosomal regions from several kilobases to several hundred kilobases [19,20,21,22,23,24], thus calling for completely new models of transcriptional regulation [12,25,26]. Among the factors so far implicated in the formation of chromosomal expression domains are strong transcription units [27,28], regulatory interactions [29], binding effects of nucleoid-associated proteins (NAPs) and other DNA structuring proteins including macrodomain-specific DNA binding proteins [30,31,32], DNA topoisomerases [25], and last but not least, the peculiar sequence organization of chromosomal regions [22,23,24].

Whatever the nature of the factors determining the subdivision of the chromosome into separate expression domains, recent studies made it obvious that the coordination of genomic function involves the temporal emergence of coherent domains of transcription (CODOs, formerly denoted as DCTs [25]) comprising large (up to megabase-sized) chromosomal regions and resembling the so-called pathogenicity islands with peculiar sequence organization, that can form “archipelagos” in the genome [33,34]. Understanding the mechanism organizing the CODOs in the genome requires an integrative (holistic) study revealing the links between the spatial patterns of gene transcription, the structural dynamics of the corresponding chromosomal regions, and the global sequence organization of genome. 

## 2. Model of Regulation of Gene Expression during the *E. coli* Growth Cycle—A Brief Overview

**Figure 1:**
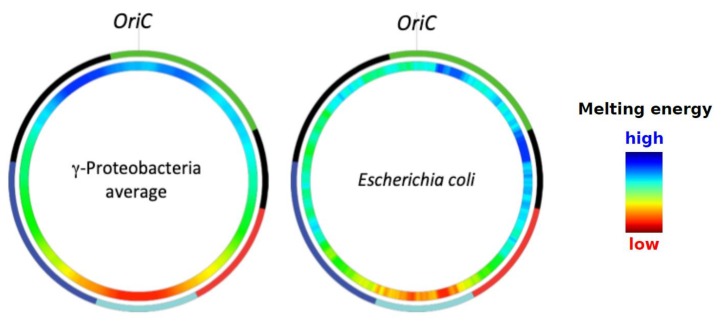
Distribution of DNA average negative melting energy in the circular genomes of γ-proteobacteria and *Escherichia coli*. Color-coded in blue and red represent, respectively, the high and low average negative melting energies of the sequences. The outer ring indicates, by color, the chromosomal macrodomains: Ori (green), Right (red), Left (dark blue), Ter (light blue), LNS (Left Non-Structured region) and RNS (Right Non-Structured region) in black. OriC: origin of replication. Scanning window size is 500 kb sliding by 4 kb. Adapted from Reference [23].

In *E. coli*, the role of chromosomal sequence organization in the regulation of the genetic function is quite conspicuous. Initiation of replication at the chromosomal origin of replication (OriC) requires high negative superhelicity both in vitro and in vivo and is assisted by several DNA architectural proteins including highly abundant NAPs, such as integration host factor (IHF), factor for inversion stimulation (FIS), and histone-like protein first isolated from *E. coli* strain U13 (HU) [35,36,37,38,39,40,41,42]. Importantly, in γ-proteobacteria in general and in *E. coli* in particular, the sequences around OriC demonstrate a higher thermodynamic stability compared to those around the chromosomal terminus (Ter) of replication (Figure 1). 

In *E. coli*, the G/C-rich OriC end is activated early on commitment of cells to exponential growth [23]. This early activation of the OriC end is associated with the activation of DNA gyrase, which introduces negative supercoils in the DNA [43] and increases the expression of supercoiling-dependent strong transcription units (transcriptons) encoding ribosomal RNA (*rrn*) and protein genes, as well as of the RpoZ (ω) subunit of RNA polymerase (RNAP), which stabilizes the vegetative (σ^70^) holoenzyme assembly [44,45,46] and the activator of *rrn* transcription FIS [46,47,48,49,50,51,52,53,54,55], among other genes involved in boosting growth. Activation of these strong transcriptons requires high negative superhelicity, supplied on the one hand by DNA gyrase, demonstrating a decreasing gradient of binding sites from OriC to Ter [19,21] and on the other hand, by increased production of negative supercoils in the wake of both the translocating replisomes and numerous RNAP molecules engaged in the transcription of stable RNA operons, all of which are oriented towards the Ter [56,57,58]. Notably, these powerful DNA translocases also increase the positive superhelicity ahead of them, which can be counterbalanced by increased gyrase activity observed downstream of the highly transcribed operons [59,60]. Although direct experimental evidence is still lacking, all these effects are thought to lead to higher negative superhelicity in the chromosomal OriC end compared to the Ter end [61], thus facilitating the utilization of thermodynamically stable (G/C-richer) DNA sequences for transcription. Indeed, in cells lacking the major NAP HU, the chromosomal rrn domain, encompassing OriC together with the most distal *rrn* operons on both chromosomal arms, is activated aberrantly on the expense of genes requiring high negative superhelicity for transcription, suggesting that HU is involved in buffering the accumulation of excessive negative superhelicity in the OriC end, which otherwise, could impair the coordinated transcription of the genome [3,21,62]. The NAP FIS, abundant during the commitment of cells to fast exponential growth, exerts a more local coordinating effect by stabilizing DNA loops at upstream activating sequences (UAS) of stable RNA operons and buffering them at deviations of superhelical density [63,64].

On cessation of replication, when the processes increasing the supercoil dynamics in the G/C-richer chromosomal Ori end subside, the less G/C-rich Ter end gains a competitive advantage in terms of energy requirements for duplex melting associated with the initiation of transcription. This switch in the relative activity of chromosomal ends is coordinated with a change in RNAP holoenzyme composition and promoter recognition specificity [65,66,67], apparently assisted by abundant NAPs, demonstrating a high frequency of repressor sites in the Ori end, such as heat-stable nucleoid-structuring protein (H-NS) and leucine-responsive protein (Lrp) [61], as well as increased expression of DNA-binding protein from starving cells (Dps), the most abundant stationary-phase NAP involved in condensing the DNA [40,68]. H-NS and Lrp also synergize to shut down stable RNA promoters on depletion of nutrients [69]. Furthermore, at this stage, the negative superhelicity of the Ter end is increased [70]. Thus, ultimately, the successive transcriptional activation of chromosomal poles during the *E. coli* growth cycle appears to be governed by coupling the static gradient of DNA thermodynamic stability with the dynamic gradient of negative superhelicity along the chromosomal OriC–Ter axis, which acts as a coordinate system [23,61]. 

Importantly, the chromosomal OriC and Ter ends also differ in terms of genetic function. The former is enriched in anabolic genes and the latter in catabolic genes [71]. More compellingly, the temporal expression of genes is tightly coupled to their spatial arrangement in the chromosome—a feature that is conserved in both the γ- and the α-proteobacteria [61,72]. Thus, in *E. coli*, the spatial order of genes along the chromosomal OriC–Ter axis and the temporal expression of genetic function is coupled with a strategic organization of sequences with distinct thermodynamic stability around the opposite chromosomal poles. This superimposition of the gradients of DNA thermodynamic stability and supercoiling on a conserved chromosomal gene order with anabolic and catabolic functions, allocated respectively to the OriC and the Ter ends, provides a bona fide device for the spatiotemporal coordination of genomic expression during the *E. coli* growth cycle [73]. The dynamic patterns of CODOs emerging during the *E. coli* growth cycle and featuring distinct sequence organization are consistent with this notion [23]. However, the genomic sequence organization in the commensal bacterium *E. coli* is likely streamlined for fast growth. The question is whether a similar organization can be revealed in related bacteria with more sophisticated lifestyles, such as, e.g., bacterial pathogens. One excellent model to explore this question is the plant pathogenic bacterium *Dickeya dadantii,* which during plant infection, has to cope with various types of severe environmental stress [74,75]. 

## 3. Model of Regulation of Gene Expression during the Pathogenic Growth of *D. dadantii*

**Figure 2:**
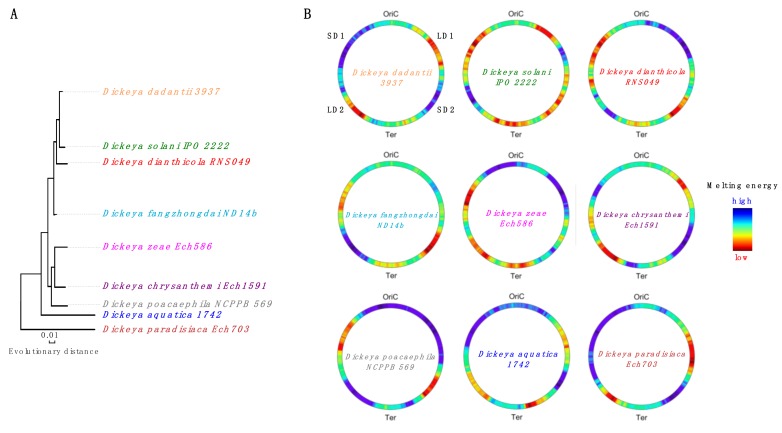
(**A**) Phylogenetic tree of the various *Dickeya* species (adapted from Reference [76]). (**B**) Distribution of DNA average negative melting energy in circular *Dickeya* genomes. The average melting energy was calculated using the parameters of SantaLucia [77] with a scanning window size of 500 kb sliding by 4 kb as in Figure 1 and Reference [24]. Color-coded in blue and red are, respectively, the high and low average negative melting energy (same color scale as in Figure 1). The G/C-rich SD1 and SD2 regions and the A/T-rich LD1 and LD2 regions are indicated in *Dickeya dadantii*. Ter: terminus of replication.

The sequence organization of *D. dadantii* genome markedly differs from that of *E. coli*. In particular, it features four (two in each chromosomal arm) thermodynamically distinct DNA regions. These regions appear to be highly conserved, although they have suffered extensive rearrangements during the evolution of *Dickeya* species (Figure 2). 

Conservation of these regions, despite the fact that some of the *Dickeya* genus representatives experienced a substantial change in their habitat, suggests that they are important for the adaptation and maintenance of pathogenicity. Indeed, recent studies in the *D. dadantii* model demonstrated that the transcriptional response of this pathogen to various kinds of stress produces coherent spatially confined gene expression patterns dubbed the “stress response” domains [24]. These latter comprise DNA sequences of different thermodynamic stability and thus closely resemble the CODOs of *E. coli.* Since these CODOs distinctly respond to alterations of DNA supercoiling, they also closely resemble the topological domains observed in the human pathogen *Streptococcus pneumoniae* [22]. 

The detection methodology of CODOs is based on exploring the correspondence between the genomic distributions of DNA analog parameters (such as gene expression density, average negative melting/stacking energy, supercoiling response of the expressed genes, preferential orientation of the transcriptons, relative frequency of binding sites for NAPs) and the distribution of digital parameters (unique genes and genetic functions) [23]. Ultimately, this methodology intends to correlate genomic sequence organization with genetic function. In *D. dadantii*, the distribution of each of these parameters was measured in the transcriptomes obtained from cells grown under distinct stress conditions mimicking the environment during pathogenic growth; subsequently, the correlations between their spatial distribution patterns were mapped in the genome to determine common, statistically significant boundaries. Analyses of transcriptomes obtained under 32 different growth conditions identified 11 different CODOs, emerging in specific constellations in response to the applied stress [24,26]. In the OriC end of the chromosome, the boundaries of some of these “stress response” CODOs closely coincide with the positions of stable RNA operons [24], in keeping with findings in *E. coli* [27]. However, the crucial question is whether it is possible to identify signatures in the basic design of genomic sequence organization that explain the dynamic behavior of the CODOs.

**Figure 3:**
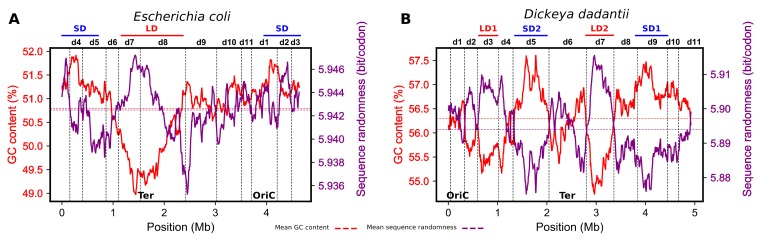
Mapping of correlations between G/C content and distributions of codons (sequence randomness) in the *E. coli* (left panel **A**) and *D. dadantii* (right panel **B**) genomes. The distributions were calculated using 500 kb windows sliding by 4 kb (as in Figure 1, Figure 2, and Reference [71]). Individual CODOs (d1 to d11) are indicated above the graphs. In *E. coli*, the CODOs d7 and d8 comprise the Ter region, and OriC is located in the middle of d1. In *D. dadantii*, the four thermodynamically distinct regions LD1, SD1, LD2, and SD2 are comprised in the CODOs d3, d5, d7, and d9 respectively. OriC is at the border between d11 and d1, whereas Ter is located in the middle of d6. The borders of the CODOs (vertical lines superimposed on the GC content/sequence randomness curves) were defined on the basis of correlation between various analog DNA information types retrieved from the transcriptomes of *E. coli* [23] and *D. dadantii* [24].

In *E. coli*, the measurements of the distribution of DNA analog (i.e., structure-related) and digital (i.e., genetic code-related) parameters of the genomic sequence revealed a strict anti-correlation between thermodynamic stability and codon variability in the OriC and Ter ends of the genome. In the G/C-rich OriC end, the randomness of codon distribution is low, whereas in the relatively A/T-rich Ter region, where thermodynamic stability is low, codon randomness is high [71]. This is because due to the degeneracy of the genetic code, G/C-rich codons are used, preferentially, in the G/C-rich region, whereas in A/T-rich regions, all the codons are used equally. Application of this approach to *D. dadantii* revealed the same regularity: the G/C-rich SD1 and SD2 regions demonstrated low sequence randomness, whereas the A/T-rich LD1 and LD2 regions demonstrated high sequence randomness (Figure 3). Furthermore, the genomic distributions of these two characteristics of DNA sequences revealed extended domains featuring an opposite skew of thermodynamic stability and codon randomness. Strikingly, these static genomic patterns largely coincided with the dynamic borders of the CODOs derived from the correlated changes of analog parameters in both the time-resolved transcriptomes of *E. coli* and the stress response transcriptomes of *D. dadantii* (Figure 3). Given the difference in the used approaches, it is highly unlikely that this coincidence is accidental. It rather suggests that not only the genetic code, but also the dynamic behavior of the chromosome is inscribed in the primary sequence organization of the genome. Importantly, while most of the CODOs comprise sequences of distinct thermodynamic stability, they also harbor specific adaptation and virulence determinants dedicated to coping with various kinds of environmental stress [24,25]. A similar organization of function in isolated gene clusters and chromosomal topological domains was observed in *Salmonella* [78], *S. pneumoniae* [62], and *Streptomyces* [23,79]. Taken together, these findings support the notion that the CODOs represent genuine sub-chromosomal structural–functional entities involved in coordinated responses of the genome to environmental change. 

## 4. Testing the Dynamical Behavior of CODOs by Reporter Constructs.

**Figure 4:**
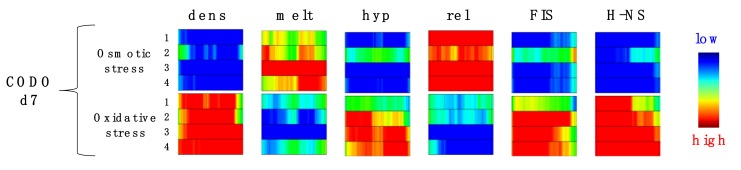
Response of the CODO d7 (560 kb) of *D. dadantii* to osmotic (upper row) and oxidative (lower row) stress. The domain is represented as a square consisting of four rectangles (numbered 1 to 4) corresponding to four different growth media. The parameters of expressed genes are color-coded (red for high, and blue for low values): dens: gene expression density, i.e., high gene density indicates a high proportion of genes upregulated, whereas low gene density indicates a high proportion of genes downregulated; melt: average negative melting energy of expressed sequences (note that unlike in Figure 1 and Figure 2, red indicates high negative melting energy, and blue indicates low negative melting energy); hyp: proportion of genes activated by high negative supercoiling; rel: proportion of genes activated by low negative supercoiling; FIS: proportion of factor for inversion stimulation (FIS)-activated genes; H-NS: proportion of heat-stable nucleoid-structuring protein (H-NS)-activated (de-repressed) genes. Adapted from Reference [24].

While we still lack a clear understanding of the mechanisms leading to their emergence, most of the CODOs feature genes that are characterized by a distinct average negative melting energy, prefer a particular supercoiling regimen, and/or coherently respond to particular NAPs. For example, the CODO d7 of *D. dadantii* comprising the LD2 region contains DNA of low thermodynamic stability (see Figure 2). Under conditions of osmotic stress, the differentially expressed genes in this CODO are mainly downregulated (Figure 4; blue square in the upper row, marked “dens” for gene expression density), whereas the majority of the still expressed genes are encoded by sequences of high average negative melting energy (red square in the upper row, marked “melt”) and activated by DNA relaxation (red square in the upper row, marked “rel”). However, on application of oxidative stress, the genes in the same CODO d7 are mainly upregulated, whereby the expressed genes are predominantly encoded by sequences of low average negative melting energy and activated by high negative superhelicity, FIS and H-NS (red columns in the lower row). 

In other words, we see that depending on the kind of challenge, the same chromosomal region produces distinct specific responses. Since the organization of CODOs reveals a combined input of several factors pertinent to the modulation of DNA supercoil dynamics, it is likely that their formation reflects changing structural dynamics of the corresponding chromosomal regions induced by environmental stress. In principle, such dynamic alterations can be examined using reporter gene constructs capable to sense regional changes of chromosomal supercoil dynamics. More specifically, the question is whether a reporter gene inserted into a particular CODO will reflect its activity (defined as the average response of its genes to a particular environmental signal). Since the emergence of a CODO is correlated with a directionally coherent alteration (either activation or repression) of gene expression, a faithful reporter construct should comply with the behavior of the CODO it is targeted to. For example, peculiar influence of the encompassing chromosomal regions on the inserted reporter cassettes has been reported in *S. pneumoniae,* where the topoisomerase genes implicated in the physiological control of supercoiling were found to be harbored by topological domains distinctly responding to DNA relaxation [80]. 

**Figure 5:**
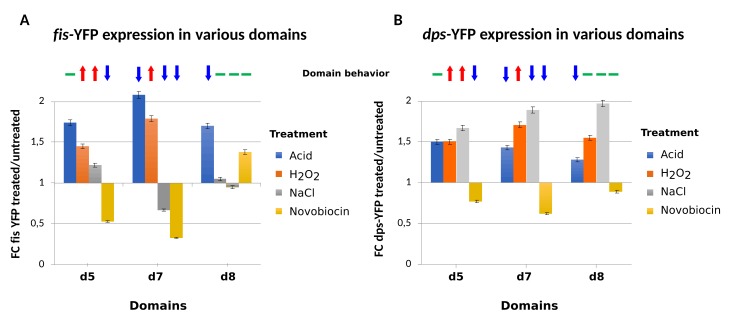
Response of reporter gene constructs to perturbations of chromosomal supercoiling. (**A**) Expression of the supercoiling-dependent *fisP*-YFP reporter construct reflects the behavior of the synchronously emerging CODOs d5 and d7 in response to oxidative and osmotic stress. The response of the same reporter in the “asynchronously” emerging CODO d8, with the exception of the response to acidic stress, is entirely different. (**B**) The response of the *dpsP*-YFP construct to perturbations is less discriminative between the domains. The domain behavior is indicated, with the red arrow corresponding to upregulation, the blue arrow to downregulation, and the green line to no change in gene expression density. The fisP-yfp and dps-yfp constructs were obtained by PCR amplification from the pUCter fis-yfp and pUCter dps-yfp plasmids [3], respectively, and contain the *E. coli fis* operon promoter sequence (comprising 300 bp upstream from the transcription start site at +1 and the downstream *dusB* ORF) and the *E. coli dps* promoter sequence (comprising 500 bp upstream from the transcription start site at +1) cloned upstream of the *yfp* gene, followed by an *rrnB* terminator and a *cat* gene conferring chloramphenicol (Cm) resistance. The PCR fragments containing the fisP-yfp-Cm^R^ and dpsP-yfp-Cm^R^ cassettes were cloned in pGEMT plasmids, electroporated in *D. dadantii* cells, and inserted in d5 (between the convergent *ousA* and ORF46989), d7 (between the convergent *fliT* and *fliE* genes), and d8 (between the tandem *pelA* and *pelE* genes) by marker-exchange recombination. The recombinants were selected in low-phosphate medium in the presence of chloramphenicol, under conditions in which pGEMT derivatives are highly unstable. Expression of the *fis*P*-yfp* or *dps*P*-yfp* fusions in different chromosomal domains was measured by a spectrofluorimeter (TECAN Infinite F200) with emission/excitation wavelengths of 548/505nm, respectively.

In *D. dadantii,* the correlation between domain behavior and reporter activity was examined using the supercoiling-sensitive *fis*P-YFP construct, which was previously shown to faithfully reproduce the regional differences in supercoil dynamics induced by deletion of HU in *E. coli* [3]. *D. dadantii* CODOs d5 and d7 emerge synchronously and harbor adaptation/virulence determinants, the expression of which significantly correlates with that of the CODOs [25]. These two CODOs respond similarly to oxidative stress but respond in opposite ways to osmotic stress. Accordingly, it was observed that the *fis*P-YFP reporter inserted in d5 and d7 demonstrates a behavior reflecting that of the targeted CODOs, i.e., activation under oxidative stress in both CODOs and activation in d5 vs repression in d7 under osmotic stress (Figure 5A). Notably, in both of these CODOs, the activity of the *fisP*-YFP reporter declined after the relaxation of DNA induced by novobiocin treatment, as expected. However, when this construct was inserted into the CODO d8, which is “asynchronous” in a sense that it does not appear in any of the examined growth conditions in combination with either d5 or d7, the reporter responded poorly to both oxidative and osmotic stresses, whereas DNA relaxation by novobiocin increased instead of decreasing the reporter activity. At the same time, the response of the *fisP*-YFP reporter to acidic stress was uniform and did not depend on the behavior of the targeted CODO. This versatile response of the *fisP*-YFP reporter markedly differed from that of the *dpsP*-YFP construct, in which the reporter is under the control of the promoter of the *dps* gene encoding Dps, the major stationary-phase NAP. In *E. coli*, this latter construct demonstrated relative independence from the chromosomal context [3] and was also much less discriminative between the domains in *D. dadantii* (Figure 5B). 

However, why does the *fisP*-YFP construct inserted in CODOs d5 and d7, as well as these CODOs themselves, respond to osmotic stress in opposite ways? Osmotic shock stimulates K^+^ uptake [81], compensated by an increase in internal glutamate concentration [82]. This change in the internal milieu could affect protein occupancy in CODOs d7 and d5, comprising sequences of, respectively, low (LD2) and high (SD2) thermodynamic stability (see Figure 2). Indeed, protein occupancy depends on both DNA composition and sequence [83,84]. Osmotic shock, besides redirecting RNAP binding in the genome [85], could also affect the binding of NAPs, which depends on both sequence composition and DNA topology [86,87,88,89,90]. For example, the binding of the global repressor H-NS [91] is modulated by ionic composition, sequence organization, and DNA topology [88,92,93,94,95,96,97]. H-NS preferentially binds A/T-rich sequences and represses transcription by bridging DNA duplexes and forming inter-wound filaments [98,99]. This bias of H-NS binding could explain the opposite effects of the osmotic shock on the G/C-rich CODO d5 and the A/T-rich CODO d7 [24], as well as the “faithful” response of the *fisP*-YFP reporter inserted into these CODOs—a conjecture that can be tested, for example, by examining the reporter behavior in the *hns* mutant strain. 

It is noteworthy that, while it is generally accepted that alterations of transcript patterns are adaptive, there are reports about the absence of correlation between the transcript pattern and the expressed function. For example, in a study comparing the expression and mutant fitness of biosynthetic genes in several bacteria including the metal-reducing bacterium *Shewanella oneidensis*, *E. coli*, the ethanol-producing bacterium *Zymomonas mobilis*, and the sulfate-reducing bacterium *Desulfovibrio alaskensis* it was reported that there is little correlation between the importance of genes for optimal growth or fitness and their upregulation [100]. Also, a recent study of the plant pathogen *Pseudomonas syringae* reported the absence of correlation between mutant fitness in planta and either the magnitude of expression or the degree of induction for most genes in planta compared to in vitro conditions [101]. However, fitness is measured cumulatively over several growth cycles, whereas expression is measured during a single growth/infection cycle, making direct comparisons difficult. In principle, the lack of strict coherence between the impact of gene mutations on fitness and the expression of the genes concerned could be explained by the existence of several genes or loci acting in parallel on the same process. The highly reproducible expression patterns of the CODOs observed under conditions of applied environmental stress or modulation of DNA topology by topoisomerase inhibitors and/or poisons suggest that functionally linked genes including virulence genes [23,24,80] are co-transcribed within a variable chromosomal context, which is beyond the detection capacity of the approaches examining the comparative fitness of individual mutants.

## 5. Conclusions

The sequence organization of the bacterial genomes reflects the necessity of facile accessibility of genomic regions encoding particular functions to regulatory factors during bacterial growth and adaptation to changing environmental conditions. We have argued that both in the commensal *E. coli* and in the plant pathogen *Dickeya,* this coordination of structure and function is achieved by the strategic organization of large chromosomal domains of gene expression containing sequences with distinct thermodynamic stability and supercoiling response, termed CODOs. The *Dickeya* CODOs and similar regions in other pathogens harbor various adaptation/virulence determinants that are co-regulated with and expressed within the CODOs, emerging in responses to environmentally induced changes of DNA supercoiling. Since global DNA supercoiling changes as a function of metabolic state, while the latter depends on growth conditions, it is likely that specific constellations of CODOs emerging in response to environmental change aid the facile adaptation of pathogenic bacteria to stress. We believe that further studies of the coupling between the physicochemical properties of DNA, genomic expression dynamics, and genetic function will pave the way towards understanding how the coordinated transcriptional response to environmental change is encoded in the primary sequence organization of bacterial genomes.
